# Future Projections of Burned Area in Europe Highlight the Importance of Human Action

**DOI:** 10.1111/gcb.71043

**Published:** 2026-08-20

**Authors:** Maik Billing, Matthew Forrest, Simon P. K. Bowring, Luke Oberhagemann, Alex N. Neidermeier, Jessica Hetzer, Werner von Bloh, Christoph Müller, Susanne Rolinski, Thomas Hickler, Kirsten Thonicke

**Affiliations:** ^1^ Potsdam Institute for Climate Impact Research (PIK), Member of the Leibniz Association Potsdam Germany; ^2^ Senckenberg Biodiversity and Climate Research Centre (SBiK‐F) Frankfurt am Main Germany; ^3^ Laboratoire des Sciences du Climat et de l'Environnement (LSCE), IPSL‐CEA‐CNRS‐UVSQ Université Paris‐Saclay Gif‐sur‐Yvette France; ^4^ Institute of Environmental Science and Geography University of Potsdam Potsdam Germany; ^5^ Environmental Geography Group, Institute for Environmental Studies (IVM) VU University Amsterdam Amsterdam the Netherlands; ^6^ Department of Physical Geography Johann Wolfgang Goethe University of Frankfurt Frankfurt Germany

**Keywords:** burned area, climate change, coupled fire‐vegetation modelling, Europe, fire weather, socio‐economics, wildfires

## Abstract

Wildfires are often a natural part of many European ecosystems, but human activities through land use, other socio‐economic factors and climate change have significantly changed how fires behave. Anthropogenic climate change is already intensifying fire‐prone weather and increasing wildfire risk across Europe with risk expected to grow sharply in coming decades. Past experience suggests that human action, such as fuel management and improved fire suppression capacity, can reduce wildfire spread and intensity. However, whether current or future efforts can counter rising risks under continued climate change remains uncertain. In this study, we assess the role of socio‐economic factors and biophysical factors in shaping future fire regimes across Europe. Using two fire models (SPITFIRE and BASE) coupled with the fire‐enabled Dynamic Global Vegetation Model LPJmL, we simulate future burned area under two socio‐economic and greenhouse gas concentration pathways (SSP1‐2.6 and SSP3‐7.0). We also run experiments where socio‐economic factors are held constant to isolate their influence. Our findings show that both biophysical and socio‐economic factors strongly affect future wildfire activity. Fire weather and fire management capacity are important drivers, while population density, vegetation shifts and land use matter more at regional scales. By the end of the century, intensified fire weather alone could increase annual burned area by approximately +39% under a low emission scenario (SSP1‐2.6) and by nearly +192% under a high emission scenario (SSP3‐7.0). Continued improvements in fire management capacity could substantially moderate these increases, reducing burned area by 72%–92% compared to scenarios without these improvements. However, under strong climate change, fire activity still rises in about 55% of Europe's fire prone regions, even if investments in fire management capacity continue at current levels. Overall, improved wildfire management capacity has the potential to greatly limit impacts of worsening fire weather, but may not fully offset strong climate change impacts.

## Graphical abstract

Our simulations with two fire models and the LPJmL Dynamic Global Vegetation Model show that both biophysical and socio‐economic drivers shape future wildfire activity in Europe. Fire weather and fire management capacity dominate broad trends, while changes in land use, vegetation and population density shape regional patterns. Improved fire management could moderate but not fully counter climate‐driven increases in fire activity under strong climate change.

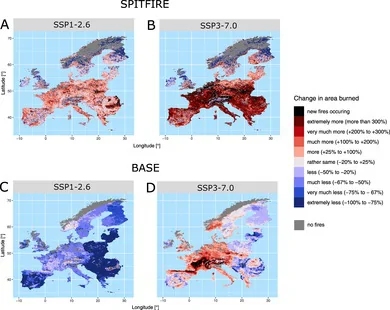

## 1 Introduction

Wildfire activity is increasing in Europe, putting both human lives and natural capital at risk (Fernandez‐Anez et al. 2021; Meier et al. 2023; Arrogante‐Funes et al. 2024). Yet fire is also a natural process with an important role in shaping biodiversity and ecosystem dynamics (Bowman et al. 2009; He et al. 2019). In Europe, centuries of human activity have significantly transformed landscapes, influencing the frequency, intensity, and spatial patterns of wildfires (Vannière et al. 2016; Galizia et al. 2021; Pausas and Keeley 2021). In the coming decades, rising temperatures and more frequent droughts due to climate change are expected to further increase the likelihood of severe fire danger across the continent (El Garroussi et al. 2024; Hetzer et al. 2024). However, wildfire dynamics are not only driven by meteorological conditions—called ‘fire weather’—but by a complex interplay of climate, vegetation, and human activities (Bowman et al. 2009; Pausas and Keeley 2021). In Europe, burned area has declined in several regions over recent decades despite increasingly favourable meteorological conditions for wildfire (Turco et al. 2016; Urbieta et al. 2019; Otón et al. 2021; Kelley et al. 2025). This apparent contradiction suggests that human interventions—such as enhanced fire prevention, suppression, land cover change and evolving land management—have played a crucial role in moderating the recent history of European fire activity (Turco et al. 2016; Bountzouklis et al. 2022). While fire suppression is often desired to protect human lives and assets, singular reliance on suppression can lead to fuel accumulation and unintended consequences for fire‐adapted ecosystems, potentially increasing the likelihood of more severe and uncontrollable fires (Kreider et al. 2024).

Fire‐enabled Dynamic Global Vegetation Models (DGVMs) have become essential tools for gaining insight to the interaction of climate, vegetation, humans, and wildfire (Hantson et al. 2016, 2020; Li et al. 2019). DGVMs simulate the effects of climate change and land use on vegetation and soils at large scales (regional to global) through integrated impacts on carbon, water and energy fluxes (Prentice et al. 2007). Fire models can be embedded in DGVMs which represent fire behaviour by incorporating fuel characteristics, weather patterns and anthropogenic drivers to project fire behaviour and effects (Teckentrup et al. 2019; Hantson et al. 2020). Changes in vegetation determine the type and amount of fuel available to burn (Curt et al. 2011; de Magalhães and Schwilk 2012). Land cover and land‐use changes, such as transitions from forest to grassland or cropland expansion, directly alter fuel characteristics. Conversely, agricultural abandonment can increase vegetation cover and fuel accumulation—a trend especially observed in fire‐prone landscapes in southern Europe (Moreira et al. 2011; Viedma et al. 2015; Sil et al. 2019). Such vegetation‐driven changes feed back into fire models, linking climate, land use, and ecosystem dynamics to fire into a single modelling framework (Thonicke et al. 2010).

The mechanics of simulating wildfire behaviour in fire‐enabled DGVMs vary widely (Hantson et al. 2020; Forrest et al. 2024). In particular, the influence of human activities is approached in different ways and has evolved substantially over time (Le Page et al. 2015; Hantson et al. 2016; Teckentrup et al. 2019). Early global fire models such as GLOBFIRM did not explicitly represent human activities, instead focusing primarily on climate–vegetation controls (Thonicke et al. 2001). Subsequent developments introduced increasingly explicit representations of human effects. In the Reg‐FIRM model, human population density was introduced as a proxy for ignition probability and tested for the Iberian Peninsula (Venevsky et al. 2002), and this approach was further developed to the global scale by Pechony and Shindell (2009) and in the original SPITFIRE model (Thonicke et al. 2010), which additionally represents suppression of ignitions under high population density to reflect reduced fire use and increased landscape fragmentation in densely populated regions. Similarly, the Canadian Terrestrial Ecosystem Model (CTEM) incorporates spatially explicit probabilities of human‐caused ignitions to capture regional variability in human access and activity (Arora and Boer 2005). In contrast, statistical approaches such as SIMFIRE (a global scale model, Knorr et al. 2014) or the recent BASE model (“Burnt Area Simulator for Europe”), human influences are directly inferred from data, while still constraining the choice of variables by process understanding and theory (Forrest et al. 2024). BASE, for example, integrates an empirical finding for Europe—that countries with higher Human Development Index (HDI) values have a greater capacity and interest to invest in fire management to mitigate their negative impacts. These two modelling paradigms—globally transferable process‐based representations and regionally constrained empirical formulations—provide complementary but distinct ways of representing human–fire interactions.

Simulations with such models are particularly valuable in the European context, where the interplay of climate, land use, and human intervention is especially complex. While recent evidence suggests that human actions can partially mitigate wildfire impacts (Turco et al. 2016; Urbieta et al. 2019; Otón et al. 2021), it remains uncertain whether these efforts will be sufficient to counter increasingly fire‐prone conditions and climate‐induced changes in vegetation (Burton et al. 2024; Synolakis and Karagiannis 2024). Previous studies have highlighted regional differences in the importance of climate, vegetation, and socio‐economic drivers for future burned area in Europe (Wu et al. 2015). Building on this knowledge, our study compares two modelling frameworks that represent human—fire interactions in fundamentally different ways: the process‐based SPITFIRE model, which uses globally applicable demographic controls on ignition and suppression, and the empirically constrained BASE model, which represents human influence through regionally calibrated socio‐economic indicators (HDI). Together with recent updates in vegetation and fire‐process representation (e.g., nitrogen limitation in LPJmL; von Bloh et al. (2018); and the updated SPITFIRE implementation at higher spatial resolution; Oberhagemann et al. (2025)), this allows a more detailed assessment of how human, climate, and vegetation factors jointly shape future fire regimes. To analyse the effect of human and biophysical factors on wildfires under climate and land‐use change, we first coupled both fire models, SPITFIRE and BASE, into the LPJmL (Lund‐Potsdam‐Jena managed Land) DGVM (Schaphoff, Forkel, et al. 2018; Wirth et al. 2024). We then applied them in a set of simulation experiments to investigate how changing driver conditions, including changing vegetation, may shape future European fire regimes under two contrasting Shared Socioeconomic Pathways (SSPs) to account for uncertainties in future climate forcing and land‐use trajectories. Finally, we conducted sensitivity experiments in which the main human drivers in those modelling frameworks—human population density, HDI and land use—were held constant. We analysed changes in simulated burned area as a proxy for future fire regimes. Simulations were carried out from 2000 to 2100 using climate input from five bias‐corrected General Circulation Models (GCMs) under a low emission scenario (SSP1‐2.6) and a high emission pathway (SSP3‐7.0), along with corresponding future land‐use change trajectories generated using CLUMondo, a spatially‐explicit land‐use model (Van Asselen and Verburg 2013; Malek et al. 2018). We discuss the influence of population density, land use, HDI, fire weather, and vegetation dynamics in our simulations. These drivers have been selected as they reflect the main mechanisms controlling fire in the models used in this study and elsewhere (Wu et al. 2015). We analyse the influence of these climatic and socio‐economic drivers and explore their role in shaping future wildfire activity in Europe.

## 2 Materials and Methods

### 2.1 Combined Fire‐Vegetation Modelling

**FIGURE 1:**
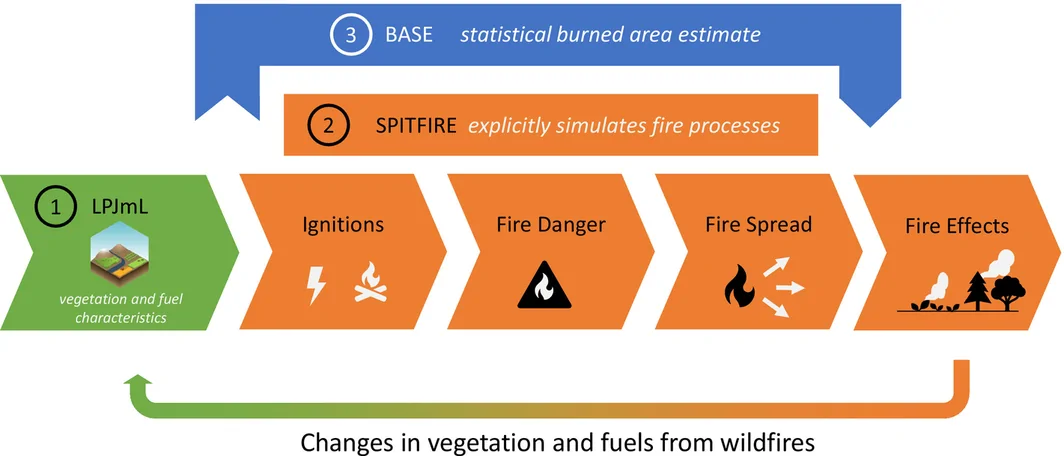
General overview of the two fire modelling approaches coupled to the DGVM LPJmL. Fire models obtain fuel properties and vegetation characteristics from LPJmL (circle 1). In the SPITFIRE model (circle 2), fires are explicitly simulated through human and lightning ignitions, danger, spread and effects which influence fuel and vegetation structure, thereby feeding back to the vegetation model (circle 1). The BASE‐integrated approach (circle 3) circumvents first three wildfire components and calculates burned area using a statistical approach. Fire effects (mortality and carbon emissions) in the BASE‐integrated approach are still calculated by SPITFIRE. The scheme was adapted from Oberhagemann et al. (2025).

This study aims to assess how climate, land use, and human intervention may shape future European fire regimes. To do so, we conducted future simulations with two alternative fire models, SPITFIRE and BASE, and analysed the simulated burned area of both models. To account for future changes in vegetation and fuel characteristics driven by land‐use and climate change, both modelling approaches were embedded for this study in the DGVM LPJmL 5.7.10 (Wirth et al. 2024). The three main modelling components—LPJmL, SPITFIRE, and BASE—are described in the following subsections (Figure 1).

#### 2.1.1 Description of the LPJmL Model

The Lund‐Potsdam‐Jena managed Lands (LPJmL) model simulates both natural and agricultural ecosystems, as well as land‐atmosphere exchange fluxes of carbon, nitrogen and water on a global scale (Bondeau et al. 2007; Schaphoff, Von Bloh, et al. 2018; von Bloh et al. 2018). The model simulates vegetation dynamics on the basis of plant functional types (PFTs) including 8 woody and 3 herbaceous PFTs (Schaphoff, Forkel, et al. 2018; von Bloh et al. 2018). Vegetation dynamics are driven by climate input data (daily temperature, precipitation and short and longwave radiation), atmospheric CO_2_ concentration, soil data (soil texture and soil depth), nitrogen depositions and land‐use fractions. A detailed overview of fundamental ecosystem processes in LPJmL, including vegetation dynamics and hydrology, is provided by Schaphoff, Von Bloh, et al. (2018), while the implementation of the nitrogen cycle is described in von Bloh et al. (2018) with latest updates in Wirth et al. (2024). Nutrient limitation influences vegetation productivity, and thus fuel production which can have non‐linear effects on wildfire. Land‐use changes affect the potential maximum area burned, which corresponds to the extent of natural vegetation and managed grasslands within a grid cell. This area may increase through land abandonment or decrease due to conversion to permanent agriculture or urban land. In this study, we allowed both fire models to burn on “natural land” (forests & natural grassland) as well as on managed grasslands. Both fire models described below were implemented within LPJmL version 5.7.10 used in this study (nitrogen & tillage version; Lutz et al. 2019; Wirth et al. 2024).

#### 2.1.2 Description of the SPITFIRE Model

**FIGURE 2:**
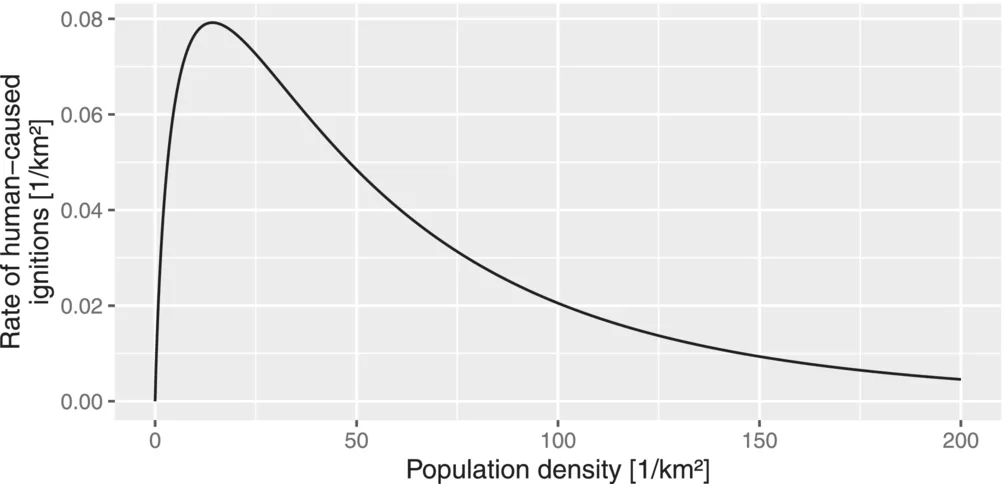
Rate of human‐caused ignitions as a function of population density in the SPITFIRE model. Ignitions rise with population density up to ~14 individuals per km^2^, then decline toward zero at very high densities.

SPITFIRE (Thonicke et al. 2010) is a process‐based fire model that can be coupled to DGVMs for vegetation‐fire simulations. SPITFIRE can simulate the ignition, spread and intensity of wildfires on a global scale (Thonicke et al. 2010). The model requires input data on fuel properties, wind speed, lightning strikes, human population density and relative humidity to calculate fire danger. In this process‐based model, wildfires originate from potential human and lightning‐caused ignitions. Human‐caused fire‐ignitions are a function of population density to differentiate between rural and more urban lifestyles (Figure 2). At low to moderate densities, ignition rates rise because fires may be started intentionally or accidentally, but they decline beyond a threshold as urbanization reduces both fire use and opportunities for accidental ignitions. Lightning ignitions are modelled using a 12‐monthly climatology that varies spatially. Potential ignitions are used in combination with a model‐calculated fire danger index to compute the proportion of ignition events, which become spreading fires. Fire danger is calculated using vapour pressure deficit (Drüke et al. 2019, replacing the original calculation based on the Nesterov Index). The resulting fire spread is calculated on the basis of the Rothermel equation (Rothermel 1972), which depends on wind speed and the availability, composition and wetness of fuel. We also implemented recent corrections to the original SPITFIRE formulation, which include (Oberhagemann et al. 2025): (1) the computation of dead fuel moisture based on the litter‐layer moisture which is now calculated for natural vegetation and has the effect to shield the top soil layer from evaporative loss (based on the tillage implementation, Lutz et al. 2019); (2) revised live fuel moisture calculation; (3) updates to fire intensity calculations; (4) adjustments to post‐fire mortality dynamics; and (5) the ability to represent multi‐day burning. Fire durations in the original SPITFIRE version were limited to a maximum of 1 day, following the assumption that larger fires and multi‐day burning can be replaced by a number of smaller fires igniting each day independently. In this study, we allowed fires to burn for multiple days. This allows fires to continue to burn into subsequent days, subject to an adjustable maximum duration. Additional stopping conditions extinguish all fires within a grid cell when the model‐calculated fire danger index falls below a certain threshold (here: 0.01), alongside a fire‐line intensity threshold (here: 5 kW/m^2^, details in Oberhagemann et al. 2025). SPITFIRE was enabled with multi‐day fires on natural land classes (natural grassland and natural forests) while single‐day fires occurred on managed grasslands. Fires on natural land were allowed to persist for a maximum of 3 days. Within each day of an active fire, the effective burning period was limited to 2–5 h to represent sub‐daily variability in fire activity. In contrast, fires on managed grassland were restricted to a single day of occurrence, with a maximum burning duration of 2 h on that day. In the coupled LPJmL‐SPITFIRE, simulated fire regimes are driven by the interplay of potential ignitions, fire weather, and fuel availability. Fire‐related tree mortality depends on simulated fire intensity over a given burned area and the type of vegetation affected. For LPJmL‐SPITFIRE simulations, we used the standard lightning and human ignition probability density function approaches as described in Thonicke et al. (2010).

#### 2.1.3 Description of the BASE Empirical Model

The BASE model (Burnt Area Simulator for Europe, Forrest et al. (2024)) is an empirical model that was developed by fitting Generalised Linear Models (GLMs) to the ESA FireCCI51 dataset (Lizundia‐Loiola et al. 2020). BASE simulates burned area over the European territory at 9 km spatial resolution, with a different set of equations for croplands and non‐croplands. Here, we used the non‐cropland version. To generate the target variable for fitting BASE (burned area), the FireCCI51 dataset was processed in combination with the ESA LandcoverCCI (ESA 2017) to derive the burned area fraction per grid cell within an aggregated land cover termed “Non‐cropland Vegetation”. This land‐cover type included all vegetation types in the LandcoverCCI classification except for croplands and cropland mosaic classes. The predictor variables used in BASE were chosen based on factors such as their relevance to fire dynamics to reflect weather, fuel, socioeconomic, or topographic conditions (Table 1, Equation (1)). During model development and fitting, the vegetation variables of tree cover, FAPAR (fraction of absorbed photosynthetically‐active radiation), and GPP (gross primary productivity) were represented using remotely‐sensed satellite data. To include the BASE model in the LPJmL modelling framework, we replaced the burned area calculation in SPITFIRE with the BASE model, thereby bypassing SPITFIRE's explicit simulation of ignitions, fire danger and fire spread. However, fire effects on vegetation remain governed by SPITFIRE's fire intensity and post‐fire mortality functions (Figure 1). Vegetation variables required by BASE (MEPI, FAPAR & tree cover) were exchanged for the LPJmL vegetation state variables to form a coupled fire‐vegetation modelling setup.

**TABLE 1:** Predictor variables used in the BASE burned area model, the reason for their inclusion, their temporal resolution, and the form of their term in the model. Adopted from Forrest et al. (2024). Variables in bold were quantified with satellite data in the model development and fitting stage, but were replaced with LPJmL model state variables in model runs presented here.

Variable	Inclusion rationale	Temporal resolution	Influence
Fire Weather Index (FWI)	Fire weather conditions contribute to the likelihood of wildfire ignition and spread	Monthly	Positive, natural logarithm and interaction with MEPI (also positive)
**MEPI (monthly ecosystem productivity index)**	Health and phenological state of vegetation to proxy live fuel moisture and dead fuel proportion	Monthly	Positive, linear, and interaction with FWI (also positive)
Human Development Index (HDI)	Socio‐economic: cultural practices, investment in firefighting, public awareness and legislation	Annual	Negative, linear
**Fraction of absorbed photosynthetic active radiation (FAPAR12)**	General fuel build‐up over last 12 months	Past 12 months	Positive, linear
Human population density (popdens)	People starting fires	Annual	Positive, linear
**Tree cover (%)**	Fuel characteristics (more fine fuel in sparse canopies) and ecosystem openness	Static map (annual in coupled runs)	Positive linear term and a negative quadratic term
Slope	Topography affects rate of spread, fragmentation, access	Static map	Positive, linear
Topographic Position Index (TPI)	Topography affects rate of spread, fragmentation, access	Static map	Positive, linear

In BASE, the Fire Weather Index (FWI) and MEPI are the first and second most important predictors (Table 1). MEPI is defined as the Gross Primary Productivity (GPP) of the month divided by the maximum monthly GPP of the last 12 months (including the current month). It is designed to capture (with low values) times when the vegetation is undergoing drought‐stress and/or is in a dormant/senescent state, both of which would indicate low live fuel moisture and a higher proportion of dead fuel and hence higher flammability (Forrest et al. 2024).

The most important socioeconomic variable in BASE is the Human Development Index (HDI), while being the third most important burned area predictor across all variables for Europe (see HDI description in Section 2.2.3). The inclusion of HDI builds on the assumption that countries with a high HDI will have the financial, institutional and human capital necessary to invest in fire management that effectively reduces burned area. HDI was found to accurately capture the historical declining trend in European burned area. Likewise, global‐scale analyses have identified HDI as a significant predictor of burned area variability (Chuvieco et al. 2021). Inclusion of HDI in BASE also led to improved simulation of spatial patterns of burned area with respect to observations (e.g., increased burning in the Balkans, details in Forrest et al. 2024). Beyond the BASE model, other fire modelling approaches also incorporate HDI or analogous socio‐economic proxies. For instance, the WHAM! model uses HDI to construct “agent functional types,” which describe how human populations manage landscapes and fires under varying levels of development (Perkins, Kasoar, et al. 2024).

BASE includes tree cover as a quadratic term because that was found to improve the model fit considerably, with the strongest enhancement of burned area at low‐to‐moderate tree cover. This is attributed to the fact that open European ecosystems (i.e., little tree canopy cover) will have a drier microclimate (De Frenne et al. 2021) and a greater amount of fine fuel in sparse canopies (grass, shrubs, lichens and mosses). The other terms had comparatively less influence (see Table 1).

(1) $$\begin{array}{c} \text{Burned area}=c_{0}+\propto_{1}\cdot \text{FAPAR}12+\propto_{2}\cdot \text{Slope}+\propto_{3}\cdot \mathrm{TPI}\\ \;+\propto_{4}\cdot \text{Popdens}+\propto_{5}\cdot \mathrm{HDI}+\propto_{6}\cdot \text{Treecover}\\ \;+\propto_{7}\cdot {\text{Treecover}}^{2}+\propto_{8}\cdot \text{MEPI}+\propto_{9}\cdot \ln\left(\mathrm{FWI}\right)\\ \;+\propto_{10}\cdot \text{MEPI}\cdot \ln\left(\mathrm{FWI}\right) \end{array}$$

Equation (1) Regression equation of the BASE model. Each of the predictors is described in Table 1. The regression was performed using a logit link function (quasi‐binomial family) over the whole European domain (for further details see Forrest et al. 2024).

### 2.2 Input Data and Simulation Protocol for Combined Fire‐Vegetation Modelling

#### 2.2.1 Climate, Soil and Nitrogen Depositions

The climate input data for our future simulations was drawn from the following five global climate models (General Circulation Models) developed via the climate model intercomparison project CMIP6 (Eyring et al. 2016): ACCESS‐CM2 (Ziehn et al. 2020), CanESM5 (Swart et al. 2019), CNRM‐ESM2‐1 (Séférian et al. 2019), EC‐EARTH3 (EC‐Earth Consortium (EC‐Earth) 2020), MPI‐ESM1‐2‐HR (Müller et al. 2018). We used bias‐corrected and downscaled CMIP6 climate projections produced within the FirEUrisk project. The five climate models were selected to represent the lower, medium, and upper ranges of projected changes in the Drought Code—a component of the Canadian Fire Weather Index (FWI) system—between the beginning and the end of the 21st century, thereby sampling a representative range of future fire weather conditions. The original grids from each dataset were bias‐corrected and downscaled to 9 × 9 km grids (see Hetzer et al. 2024) covering Europe, between latitudes 32° N and 72° N and longitudes between 15° W and 35° E. The climate model data were bias‐corrected to ERA5‐LAND climate data (see Hetzer et al. 2024), which were also used to perform historical validation simulations with both fire models. Soil texture and soil acidity (pH) were derived from the Harmonized World Soil Database (HWSD) version 1.2 (Nachtergaele et al. 2012) and processed to the 9 × 9 km grid by using the LandInG toolbox (Ostberg et al. 2023). Soil depth was set to 2 m for each grid cell. Nitrogen depositions (dry NH_4_ and NO_3_) were taken from ISIMP3 project (Tian et al. 2018) which were based on CMIP6 forcing data (Hegglin et al. 2016, 2018a, 2018b). The deposition fields were regridded from the ISIMIP 0.5° spatial resolution to the ~9 × 9 km grid by assigning to each finer grid cell the value of the coarser grid cell in which its centre is located. Nitrogen depositions were applied on a daily step to the corresponding mineral nitrogen pools of the model's topsoil layer as described in von Bloh et al. (2018).

#### 2.2.2 Land Use Input

A harmonized land‐use data set was developed for the 1960–2050 period using historic land‐use data from the HILDA+ dataset (Winkler et al. 2021) and future land‐use simulations from CLUMondo. CLUMondo, a spatially explicit land system model (Van Asselen and Verburg 2013; Dou et al. 2023), allocates demand for resources (e.g., wood, crops, livestock) which are derived exogenously from global models (here, the partial‐equilibrium model GLOBIOM, Integrated Biospheres Futures (IBF) (2023)). The change in demand is spatially allocated in CLUMondo based on spatial characteristics, local suitability, and conversion restrictions at yearly timesteps. Beyond 2050, land use fractions were held constant until the end of the century. For consistency with the LPJmL land‐use scheme, input fractions of forests, shrublands, and bare soils were merged into LPJmL's “natural land” class, while grassland fractions were assigned to managed grasslands in LPJmL. All other land uses are considered non‐burnable and masked from the model output. Each pixel thus has a percentage of land that can burn, and this fraction changes over time according to the land‐use input.

#### 2.2.3 Human Development Index

The Human Development Index (HDI) is a compound‐variable measure that seeks to proxy the relative well‐being of people across locations considering life expectancy, education and per capita income (Stanton 2025). Globally, in 2023, countries with very high human development recorded HDI values of around 0.97, while those with low human development had values near 0.4 (Stanton 2025). The HDI input was used in the LPJmL‐BASE modelling approach. Historical HDI data were produced by Kummu et al. (2018), who used the same native five arc minutes resolution (0.0833°, ~9 km) as in this study. Future projections (from 2015 onwards) were taken from (Perkins, Saxena, et al. 2024), which demonstrated continuity with historical data (Figure S8).

#### 2.2.4 Human Population Density

Historical human population density was retrieved from Perkins, Kasoar, et al. (2024); Perkins, Saxena, et al. (2024) ranging from 1960 to 2017. Human Population density projections were adapted from the 2020–2100 1‐km gridded population density estimates produced by Wang et al. (2022). The original data were downscaled to half degree and ~9 km resolutions using a conservative interpolation technique remapcon in the Climate Data Operators (CDO) software (Schulzweida 2022). Conservative remapping is considered more suitable for interpolating a discontinuous data structure, as is often the case for population data. The time‐continuous yearly series was generated by assuming a linear, equal‐annual‐increment change in the differential of the pixel value between two 5‐year time steps in the original data file. To interpolate that data gap between the historical dataset (last year 2017) and the projections (starting in 2020), we also assumed a linear incremental change between the two datasets.

#### 2.2.5 Scenarios

All input data—including climate, population density, HDI, and land use—were prepared for two contrasting scenarios, which are referred to as SSP1‐2.6 and SSP3‐7.0. Scenarios describe potential future developments based on assumptions about socioeconomic trends, policy, and climate. SSP1‐2.6, referred to as “Sustainability,” represents a socioeconomic trajectory characterized by strong mitigation efforts, including substantial reductions in greenhouse gas emissions, increased deployment of renewable energy technologies, and implementation of policies promoting sustainable development. This pathway is associated with the low‐emission climate scenario RCP2.6, which assumes strong mitigation measures and global efforts to limit radiative forcing. Under SSP1‐2.6, European population is projected to increase overall, particularly in northern and western Europe, with a preference for village and peri‐urban living. Agricultural systems shift toward more diverse and sustainable practices, and policies favour natural landscape protection and ecosystem restoration. Regional productivity diverges, with declining crop yields in southern Europe and increasing productivity in northern and western regions, contributing to spatial shifts in agricultural activity and forest regrowth.

In contrast, SSP3‐7.0, referred to as “Regional Rivalry,” represents a trajectory with slower economic growth, less global cooperation, mixed energy production from fossil fuels and renewables, and an overall low ambition to reduce carbon emissions. This scenario is coupled with the higher‐emission climate pathway RCP7.0. Population is projected to decline overall, particularly in southern and eastern Europe, accompanied by increased rural depopulation. Agricultural and livestock production decrease in several regions, especially in the south, while remaining agricultural activity becomes more polarized, with larger and more conventional farming systems dominating. These dynamics contribute to regionally heterogeneous land‐use changes, including agricultural abandonment in some areas and localized intensification in others.

Using those two contrasting scenarios allows assessment of how variations in mitigation efforts, energy use, demographic change, and land use influence modelled results. The scenarios influence land‐use patterns and population densities differently (Figures S3, S4, S9): Under SSP1‐2.6, a higher demand for forest resources increases forest regrowth in Eastern Europe along with declining agricultural activity. In contrast, SSP3‐7.0 exhibits a more heterogeneous pattern of land‐use change, without a clear overall trend. Both scenarios show increasing urbanization and declining rural population densities, with this effect being more pronounced under SSP3‐7.0.

#### 2.2.6 Simulation Protocol

For each of the five climate models, we performed a spin‐up of the natural vegetation by starting the simulation from a bare, non‐vegetated state. The simulations were run for 2500 years, looping over input forcing data for the years from 1960 to 1989. Thereafter, we conducted a 390‐year land‐use spin‐up to account for land‐use change legacy. After those two spin‐up phases, we run the simulations from 1960 to 2014 (historical period of each climate model), which was the starting point for the future simulations to diverge into the two SSPs (SSP1‐2.6 and SSP3‐7.0) scenarios. Future simulations were conducted running the models until 2100. For all simulations, the effects of nitrogen limitation on plant growth were enabled. This simulation protocol was conducted for the SPITFIRE and BASE models separately. To isolate the impact of the main drivers of both modelling approaches, we ran separate simulations for each SSP, in which HDI, population density, and land use were individually held constant from the year 2000 onward (factorial experiments, Table 2). This approach allows us to isolate the contributions of these drivers on simulated fire regimes. In both models, land use determines the extent of burnable area. Comparing these factorial experiments therefore helps to clarify how different socioeconomic factors may shape future wildfire patterns across Europe. Climatic drivers (e.g., temperature, precipitation, wind) and vegetation dynamics were allowed to vary in every simulation, as holding them constant was considered unrealistic in a fire‐enabled DGVM where climate continuously affects growth, fuel availability, and fire behaviour. The influence of fire weather and vegetation changes can be assessed indirectly through comparisons between the different SSP scenarios. To evaluate model performance, we performed historical reference runs for each modelling approach (SPITFIRE and BASE, see below) using ERA5‐LAND climate input data (see Table 2). Simulated burned area of those reference runs were compared against the GFED4s burned area product between the years 2003–2014 (Randerson et al. 2017). The reference runs captured the interannual variability in burned area reasonably well compared to observations, but spatial patterns were underestimated in Portugal and overestimated in southern Spain (see Supporting Information, section 1.1). All three models have been extensively validated in previous studies (Schaphoff, Forkel, et al. 2018; von Bloh et al. 2018; Forrest et al. 2024; Oberhagemann et al. 2025).

**TABLE 2:** Overview of the simulation experiments conducted in this study. All simulations were run for two Shared Socioeconomic Pathways (SSP1‐2.6 and SSP3‐7.0). The standard runs (SPITFIRE and BASE) allow all drivers—population density, land use, HDI, fire weather, and vegetation—to vary, representing fully dynamic fire regimes. Factorial experiments (S‐* and B‐*) hold individual socioeconomic drivers constant to isolate their effects and assess how they influence projected wildfire activity in SPITFIRE and BASE. Climatic drivers (e.g., temperature, precipitation, wind) and vegetation were allowed to vary in all simulations, as fixing them was considered unrealistic in a fire‐enabled DGVM. SPITFIRE‐hist and BASE‐hist simulations were used to compare model performance with observations.

Experiment (short name)	Description	Intention
SPITFIRE	Coupled LPJmL‐SPITFIRE with all drivers varying	Fully dynamic simulation capturing combined effects of climate, vegetation, land use, and human factors
BASE	Coupled LPJmL‐BASE with all drivers varying	
S‐Pop	Coupled LPJmL‐SPITFIRE with constant populations density from year 2000	Isolate the effect of human‐caused ignitions using SPITFIRE
S‐LU	Coupled LPJmL‐SPITFIRE with constant land use from year 2000	Isolate effect of changing potential burnable area in natural vegetation and on managed grassland in SPITFIRE
B‐Pop	Coupled LPJmL‐BASE with constant populations density from year 2000	Isolate population density effect on burned area in BASE
B‐LU	Coupled LPJmL‐BASE with constant land use from year 2000	Isolate effect of changing potential burnable area in natural vegetation and on managed grassland using BASE
B‐HDI	Coupled LPJmL‐BASE with constant HDI from year 2000	Isolate assumed effect of fire management capacity via changing HDI in BASE
SPITFIRE‐hist	Coupled LPJmL‐SPITFIRE forced with historical reanalysis climate data	Model comparison with observations
BASE‐hist	Coupled LPJmL‐BASE forced with historical reanalysis climate data	

### 2.3 Data Processing

We carried out the data evaluation using R version 3.6 (R Core Team 2019). We firstly averaged the outputs of all simulations across the five GCMs to smooth inter‐model fluctuations in the climate input data and identify climate‐driven consistent trends. To analyse changes in fire regimes over time, we first calculated the annual total area burned across the entire simulation domain for each simulation. We then computed temporal averages of the annual burned area for two distinct time periods: 2000–2030 (present‐day, hereafter) serving as the baseline, and 2070–2100 (late‐century, hereafter), again for each simulation. We then assessed the relative change in average burned area between these two periods to quantify the expected shift in fire activity under future conditions.

To identify areas with no substantial fire activity, we classified all grid cells with an average annual burned area below 0.01 ha as wildfire‐free. Furthermore, we identified grid cells with newly emerging fire activity by applying a threshold: a cell was considered to have newly occurring fires if its average annual burned area was less than one hectare during the present‐day but increased by more than 300% in late‐century. To evaluate changes in the key input variables—population density, FWI, land‐use change and HDI—we calculated and compared their temporal averages for present‐day vs. late‐century timeframe. Because land‐use change was prescribed only until 2050 and held constant thereafter, relative changes in land use were assessed using decadal averages for 2000–2010 and 2040–2050, rather than 30‐year averages as used for the other drivers and results. We then analysed the relative changes between these periods to understand how socio‐economic and climatic drivers evolved over time. We additionally computed Pearson's correlation coefficients between annual burned area and fire weather index (FWI), vegetation carbon, and fuel load for each General Circulation Model (GCM) separately. Correlations were calculated over the full simulation period, using all annual values at each grid cell for all GCMs. The resulting correlation coefficients were then mapped to analyse their spatial patterns. We also quantified the fraction of the total simulation domain exhibiting increasing and decreasing burned area based on relative changes. For the simulations in which input variables were held constant, we also calculated the annual total burned area and evaluated the differences in fire activity between the two periods, allowing us to isolate the influence of each input variable on the projected fire regimes. We quantified inter‐model uncertainty in projected burned area change using the ensemble of GCM simulations for each grid cell and SSP. For each GCM model outputs were first assigned to an ordered set of change classes representing progressively decreasing to increasing burned area change magnitudes (see Figures S18 and S19). To assess agreement among models, we converted the classes into an ordinal scale and calculated the standard deviation across the five GCMs at each grid cell (Figure S20). This metric captures the spread of projected change classes, with higher values indicating stronger disagreement among models and lower values indicating higher consistency. The resulting standard deviation fields were mapped for each SSP to identify spatial patterns of inter‐model uncertainty in projected burned area change.

## 3 Results

### 3.1 Burned Area Projections Across Models and Scenarios

**FIGURE 3:**
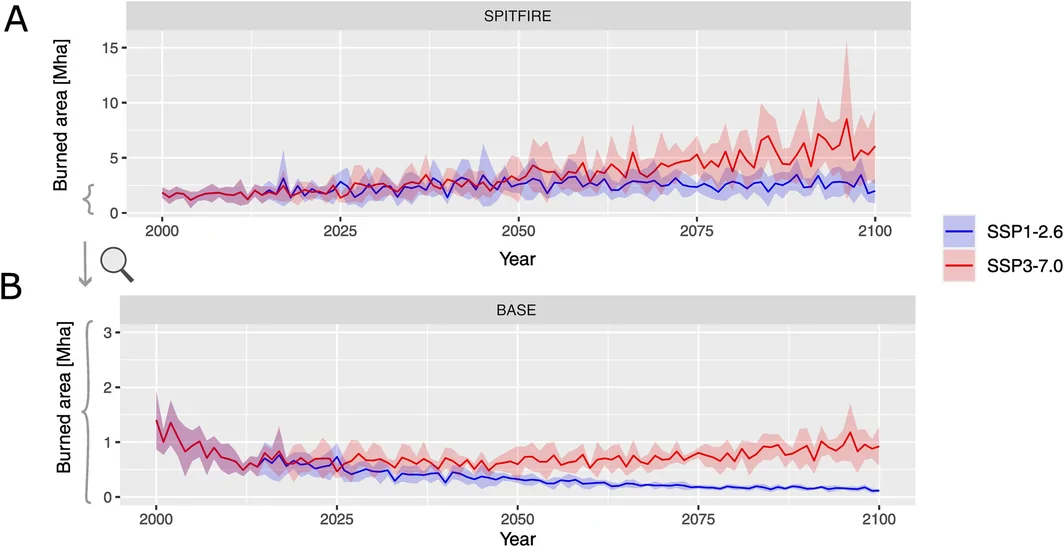
Annual sum of burned area averaged over all future projections (applying in total 5 bias‐corrected GCMs) simulated by SPITFIRE (Panel A) and BASE (Panel B) for scenario SSP1‐2.6 (blue) and SSP3‐7.0 (red). Error margins illustrate the standard deviation across all GCMs. Please note different y‐axes on panel B spanning a much lower range in burned area (depicted by curly bracket in Panel A).

Both fire models projected an increase in future burned area under the high emission scenario (SSP3‐7.0) while showing opposite trends under the low emission scenario (SSP1‐2.6; Figure 3). SPITFIRE projected a steady increase in burned area in Europe, rising from approximately 1.8 million hectares (Mha) at the beginning of the century to ~2.6 Mha (+39%) under SSP1‐2.6. Under SSP3‐7.0, the increase in burned area projected by SPITFIRE was even more pronounced, reaching ~5.3 Mha (+192%) in total (Figure 3A) which corresponds to an increase of ~3.5 Mha compared to the present.

In contrast, BASE projected a decrease in annual burned area until the middle of the century for both SSPs (Figure 3B). Under SSP1‐2.6, simulated annual burned area declined continuously, whereas under SSP3‐7.0, it increased again towards the end of the century but remained at the same level (ca. +0.06 Mha) compared to present conditions.

**FIGURE 4:**
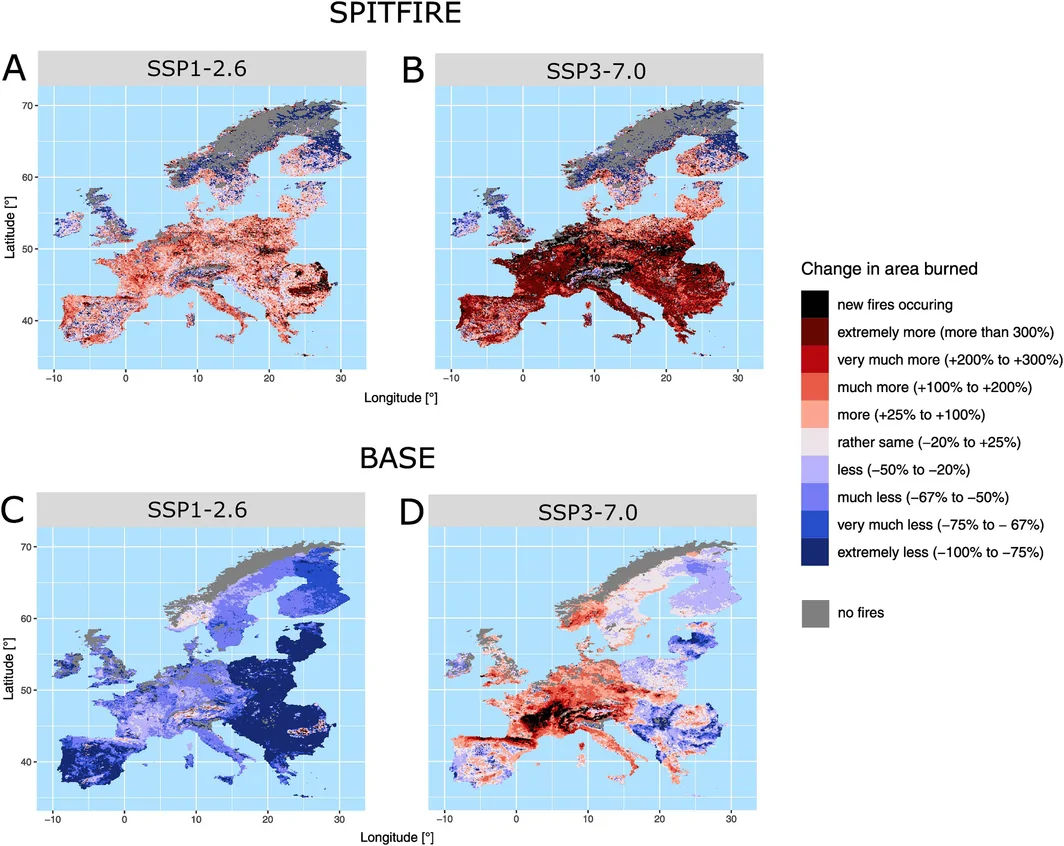
Relative changes in simulated burned area between years 2000–2030 and 2070–2100 for SSP1‐2.6 (panel A, C) and SSP3‐7.0 (panel B, D) using SPITFIRE (panel A, B) and BASE (panel C, D). Changes in burned area are displayed on a log‐symmetric scale so that increases and decreases of equal strength are comparable. For example, a + 100% rise (doubling) corresponds to a −50% drop (halving), representing equal‐magnitude changes in opposite directions.

Using the process‐based SPITFIRE model, relative burned area increased from +25 to +300% in most European regions by the end of the 21st century across all scenarios (Figure 4A,B). Under SSP1‐2.6, regions experiencing substantial fire activity today, including Greece, the western Balkans, and much of the Iberian Peninsula, are projected to experience only minor changes or even declines in burned area (Figure 4A). On the other hand, large increases in fire activity (+200% to +300%) was simulated in the Wallachian Plain, the Pyrenees and some isolated areas in Central Europe. In contrast, under SSP3‐7.0, SPITFIRE projected widespread increases in fire activity, with burned area expansions in much of Southern and Central Europe of +200% to +300%, and higher. In formerly less fire‐prone regions, SPITFIRE projects the emergence of recurrent fire regimes (e.g., Western Germany, The Netherlands). Especially, hotspots of increasing burned area under SSP3‐7.0 are concentrated in foothills and medium‐elevation areas, such as the Alps, Pyrenees, Massif Central, Sierra Nevada, Carpathians, and the Balkan and the Scandinavian ranges. The patterns align with increasing FWI under changing climates (Figure S5).

The BASE model projected substantially different spatial patterns of future fire activity. Under SSP1‐2.6, burned area decreased substantially across most of Europe, with reductions ranging from −50% to −100%, compared to present day baseline (Figure 4C). Strongest reductions were projected in Eastern Europe and parts of the Iberian Peninsula. Increases in burned area and the emergence of fires were projected in isolated areas in the Alpine foothills and parts of the Wallachian Plain. Under SSP3‐7.0, BASE projections showed more widespread burned area increases, particularly in central and western Europe with the largest increases in central France and along the Alpine foothills (+200% to +300%; Figure 4D). Despite these strong increases, parts of Eastern Europe and the Iberian Peninsula were projected to experience decreases in burned area. In total, about 55% of the simulated area showed an increase in burned area (relative change > 0) under SSP3‐7.0 (Figure 4D). Under both SSPs, BASE projected a greater reduction in burned area in Eastern Europe, the Iberian Peninsula and Eastern Europe compared to other regions. Overall, the patterns simulated by BASE are broadly aligned with country‐level regions and mirror the granularity of the HDI (Figure S7).

Inter‐model uncertainty in projected burned area change shows clear spatial structure but is not fully consistent across modelling approaches (Figure S20). Both models tend to show relatively lower agreement in alpine and boreal regions, while higher agreement is generally found in parts of the Mediterranean region, although the strength of this pattern varies. Under SSP3‐7.0, high uncertainty is observed in Central and Eastern Europe and parts of the British Isles, indicating reduced agreement among GCMs under stronger climate forcing. In contrast, SSP1‐2.6 shows higher agreement in Eastern Europe in the BASE simulations. However, this pattern is not consistent across modelling approaches, as SPITFIRE‐based projections show higher uncertainty in the same region under SSP1‐2.6. Overall, inter‐model agreement varies spatially and depends on both region and model configuration, with notable differences emerging particularly in Eastern Europe depending on scenario and fire model.

### 3.2 Simulations With Constant Drivers

**FIGURE 5:**
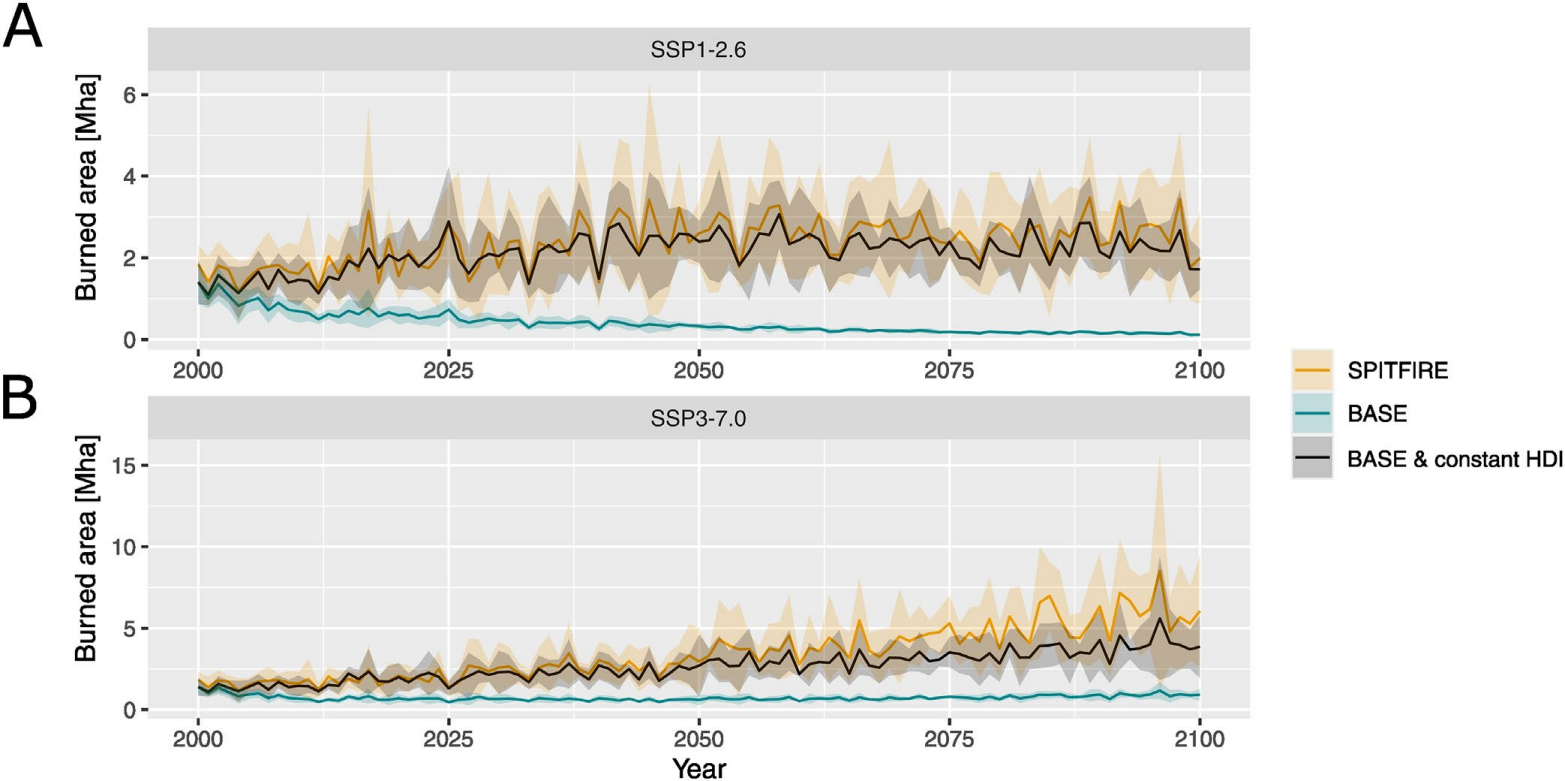
Annual sum of burned area averaged over all future projections (applying in total 5 GCMs) simulated by SPITFIRE (yellow), BASE (turquoise) and BASE with constant HDI from year 2000 (black). Panels illustrate scenario SSP1‐2.6 (Panel A) and SSP3‐7.0 (Panel B). Error margins illustrate the standard deviation across all GCMs. If HDI is held constant the BASE model projects annual burned area that closely resembles those of SPITFIRE under SSP1‐2.6. Under SSP3‐7.0 BASE with constant HDI projects similar burned area compared to SPITFIRE in the first half of the century, but substantially lower thereafter. End‐of‐century burned area is about 92% (SSP1‐2.6) or 72% (SSP3‐7.0) lower if HDI is allowed to vary (turquoise) compared to simulations with constant HDI (black).

When HDI is held constant (simulation B‐HDI, Table 2), the BASE model simulated annual burned areas similar to those simulated by SPITFIRE under SSP1‐2.6 (Figure 5A). Under SSP3‐7.0, BASE with constant HDI yielded comparable burned areas to SPITFIRE during the first half of the century, but markedly lower values thereafter (Figure 5B). By the end of the century, burned area was reduced by approximately 92% (SSP1‐2.6) and 72% (SSP3‐7.0) when HDI is allowed to vary, relative to simulations with constant HDI (Figure 5). At the regional scale, constant HDI produced widespread increases in burned area across Europe under both scenarios, while largely removing the previously observed east–west and north–south gradients in burned area reductions (Figure S14). Under SSP1‐2.6, fixing HDI yielded burned areas that are comparable to present‐day levels (−20% to −25%) in some regions (central and eastern Europe, Scandinavia), while other regions experienced increases of up to 100% with particularly pronounced increases in the Wallachian/Romanian Plain (Figure S14C). Under SSP3‐7.0, elevated burned area changes persisted in central and western Europe and parts of southern Europe in both constant‐ and variable‐HDI simulations (Figure S14B,D).

Holding land use constant (simulations B‐LU & S‐LU, Table 2) had little effect on pan‐European annual burned area in either model (Figure S12). However, land‐use change exerted pronounced regional effects under SSP1‐2.6: holding land use constant removed the projected increases in southern Spain and in the Wallachian/Romanian Plain that were formerly observed in simulations including land‐use change (Figures S16 and S17, panels A and C). Under SSP3‐7.0, regional burned area responses remained largely unchanged between simulations with fixed and evolving land use (Figures S16 and S17, panels B and D).

Holding population density constant resulted in only minor changes in pan‐European annual burned area (Figure S12). However, SPITFIRE showed greater sensitivity to population density, particularly in Central Europe, where keeping population density constant led to a spatial smoothing of burned area patterns (Figure S15).

## 4 Discussion

The simulation future changes in our study are shaped by several key drivers, including fire weather, the Human Development Index (HDI), population density, land use and vegetation (for overview see Table 3 and Supporting Information). Below, we examine how these factors contribute to variations in simulated burned area by discussing factorial simulations in which individual fire drivers were held constant from 2000 onward (see Table 2 and Supporting Information 1.5) and comparing model output from each run, as well as through relative changes in each driver from the beginning to the end of the century (Supporting Information 1.2). We subsequently also discuss how our results compare to future projections of other studies.

**TABLE 3:** Summary of key drivers of fire activity in the BASE and SPITFIRE, their relative influence, and main conclusions. Fire weather is the dominant positive driver in both model setups. HDI shows a strong negative influence in BASE and may offset fire under moderate warming. Population density primarily affects fire through ignition processes, while land use exerts smaller, spatially variable effects. Vegetation changes are particularly important in SPITFIRE through fuel composition. Further details and discussion can be found in the respective sections of the main text and Supporting Information, with supporting figures listed in the final column.

Driver	Influence in fire model		Main conclusion	Figures supporting conclusion
	BASE	SPITFIRE		
Fire weather	Strong positive	Strong positive	Dominant driver, substantial under strong climate warming	Figures 4, S5, S14, S21 and S22
HDI	Strong negative	—	Could partially offset increased fire activity, if translated into effective fire management	Figures 4, 5 and S7
Population density	Weak	Moderate	Mainly through fire ignition effect	Figures S9, S12, S13 and S15
Land use	Small continental, regional effects	Small continental, regional effects	Secondary driver	Figures S3, S4, S13, S16 and S17
Vegetation changes	Weak	Strong	Important through fuel composition	Figures S10, S11, S21 and S22

### 4.1 Fire Weather

Changes in fire weather (FWI) emerge as a dominant driver in our simulations. Under SSP1‐2.6, moderate increases in FWI occur in many regions, while others experience little change (Figure S5); by contrast, SSP3‐7.0 produces a consistent rise in fire weather index across virtually all of Europe. Under this strong‐warming scenario, escalating fire weather conditions come to dominate fire regimes throughout the Mediterranean basin and across much of France, the Pyrenees, and the western Alps (Figures 4B,D and S5).

Fire weather exerts a strong influence on burned area projections in both models. SPITFIRE shows widespread increases in burned area across Europe, even under low warming (Figure 4A), with much larger increases under SSP3‐7.0 (Figure 4B). The BASE simulations with constant HDI fire activity increase moderately under SSP1‐2.6 and substantially under SSP3‐7.0 (Figure S14C,D). These results demonstrate that fire weather also exerts a strong influence in BASE once the dampening effect of HDI is excluded. This sensitivity is expected, as FWI is the most important predictor of burned area in BASE (Forrest et al. 2024), directly capturing meteorological controls on ignition and spread. In SPITFIRE, fire weather determines fuel moisture and thus the probability of fire spread through the Rothermel equation, which explicitly depends on fuel dryness and wind speed. Consequently, both models respond strongly to changes in fire weather, although through different formulations. Additional correlation analyses support this interpretation: annual burned area exhibits consistently strong positive correlations with annual FWI across most of Europe (generally exceeding ~ +0.5, see Figures S21 and S22), whereas correlations with vegetation carbon and fuel load are substantially weaker and more spatially variable. This indicates that, at the continental scale, interannual variability and long‐term changes in burned area are more strongly associated with fire weather than with vegetation dynamics. This pattern is consistent across both SPITFIRE and BASE, although the strength of the response varies regionally. In particular, projected burned area changes across GCMs (Figure S19), reflecting greater sensitivity to the choice of climate forcing. Greater sensitivity was found in Central and Eastern Europe compared to the Mediterranean, which indicates that our results should be interpreted with greater caution in Central and Eastern Europe.

Overall, this suggests that climate‐driven fire weather is a robust driver of future fire activity across both modelling approaches. This link between fire weather and burned area strengthens confidence that continued climate warming will exacerbate fire risk, even in regions with historically low fire activity underscoring the importance of limiting climate change to reduce fire risk in Europe.

### 4.2 Human Development Index (HDI)

HDI, which is only included in BASE, emerges as a particularly strong negative predictor of fire activity, likely reflecting a link between rising human development and improved wildfire prevention, detection, and suppression capacity in Europe (Forrest et al. 2024). When HDI varies in accordance with SSP scenario's spatial and temporal changes, its strong impact on burned area becomes apparent across several European sub‐regions. In Sweden and Finland, rising HDI under both SSP1‐2.6 and SSP3‐7.0 (Figure S7) results in substantially reduced burned area (Figure 4C,D). In Eastern Europe, HDI increases under SSP1‐2.6 with substantial fire suppression, and under SSP3‐7.0, they may even offset deteriorating fire weather conditions (Figures S7, 4C,D). A similar pattern appears across much of Europe, where modest HDI improvements under SSP1‐2.6 help to reduce burned area. However, under SSP3‐7.0, this effect is often overwhelmed by more intense fire weather: HDI gains under SSP1‐2.6 (Figure S7) were found to counteract fire activity in many areas (e.g., on Iberian Peninsula, Figure 4C), HDI increases are smaller under SSP3‐7.0 (Figure 4D) and therefore provide less damping. As a result, fire regimes are increasingly shaped by more severe fire weather rather than by socioeconomic improvements. This suggests that rising human welfare could only help to offset increasing fire risk up to a certain climatic limit.

Surprisingly, we find that when HDI is held constant, the BASE model projects very similar annual burned area compared to SPITFIRE under SSP1‐2.6 (Figures 4 and 5). Although the striking convergence between BASE simulations with constant HDI and SPITFIRE projections in Figure 5 illustrates the weight of socio‐economic well‐being (HDI), we caution against over‐interpreting this as complete equivalence since model design differences between SPITFIRE and BASE remain.

Although the strong effect of HDI in the BASE model, there are important limits to how far this can be extrapolated into the future. The damping effect of HDI is based on historical data analysis and was included in the BASE model to capture the overall declining trend of burned area in the past (Forrest et al. 2024). Under the assumption that an increase in HDI means that governments and related institutions will invest in fire management to reduce fire risk in the same way, it could contribute to reduce burned area under moderate future climate conditions (SSP1‐2.6). Yet, whether the extent of historical HDI effects on fire activity can be extrapolated to a future with substantially elevated fire risk levels is unclear. That is, there may be future fire weather conditions under which fire activity cannot be dampened by HDI‐proxied socio‐economic factors: Especially extreme fire weather conditions often make fire behaviour more unpredictable and can easily overwhelm firefighting efforts (San‐Miguel‐Ayanz et al. 2013; Fernandes et al. 2016). The latest fire season of 2025 has illustrated this. In spite of high preparedness, the very extreme weather conditions in Spain lead to a record burning of nearly 400.000 ha.^1^ On the other hand, HDI can only indicate broad effects, while context‐specific, detailed and process‐based analysis is needed if one wants to capture the diversity of policy and institutional responses in regional decision‐making. Mediterranean countries have demonstrated an ability to reduce fire impacts beyond what might be expected from their HDI alone, through targeted investments, coordinated suppression strategies, and innovative management practices (Turco et al. 2016; Fernandez‐Anez et al. 2021). In addition, the strong dampening effect of HDI may be underpinned by technological innovations in satellite‐based monitoring and AI‐supported surveillance which may support early fire detection and improve fire management (Chuvieco et al. 2020; Saleh et al. 2024). However, fire detection may not always be the main bottleneck for fire management. In practice, effective suppression often depends on more basic capacities such as timely deployment of fire‐fighting crews, availability and maintenance of equipment, skilled personnel, and coordination across agencies (Marshall et al. 2022; McCarthy et al. 2023; Arango et al. 2024). These low‐tech but labour‐intensive aspects of fire management may stay challenging even in high‐HDI contexts, while they may prove increasingly difficult as fire weather intensifies. Therefore, while higher HDI can enhance societal resilience, it is not a guarantee that suppression capacity will keep pace with rising fire risk. Last but not least, unforeseen crises that disrupt societal or political developments could significantly alter trajectories of population growth future well‐being as represented by the HDI—a risk that current available scenarios likely understate by assuming smooth economic convergence without major disruptions (Buhaug and Vestby 2019). Such disruptions could weaken the link between socio‐economic development and investments in fire management capacity, particularly in a context of increasing systemic risks and potential future polycrises (Oecd and Ocde 2011; Cruz‐Castro and Sanz‐Menéndez 2016). As a result, higher levels of development would not necessarily translate into stronger fire prevention and suppression. Although historical evidence suggests that rising human development has often been associated with stronger fire management in Europe (Carlucci et al. 2019), these gains have depended on deliberate policy choices, institutional capacity, and sustained investment rather than development alone. Future fire activity will therefore depend not only on socio‐economic trajectories, but also on whether governance and fire‐management systems adapt fast enough to preserve effectiveness under climate change, fiscal constraints, and competing political priorities. Taken together, HDI should therefore be interpreted less as a guarantee of reduced fire activity and more as an indicator of the *potential* for effective fire management. This potential was found to meaningfully limit fire impacts under low to moderate climate change, but it is increasingly likely to be exceeded under severe fire weather conditions associated with stronger climate change.

### 4.3 Population Density

Population density impacted fire differently in each modelling approach. Holding population density constant in factorial BASE simulations (simulation B‐pop, Table 2) had only a minimal impact on simulated burned area (Figure S13, purple lines) relative to the full‐variation runs (Figure S13, black lines), suggesting that HDI plays a more dominant role than population density in capturing human influence within the BASE framework. By contrast, SPITFIRE demonstrated greater sensitivity to changes in population density (Figure S15). This suggests that variations in simulated human‐caused ignitions, driven by shifts in population density, are one key driver of simulated fire dynamics in Central Europe in SPITFIRE. When population density is allowed to vary, SPITFIRE projects higher burned area in Central Europe (Figure S15). This is because decreasing population density across currently highly populated areas of Central Europe increase human‐caused ignitions. This occurs because the peak in SPITFIRE's ignition probability is reached at intermediate population densities (~14 individuals per km^2^; see Figure 2), which is far below current densities in much of Central Europe. As population density declines toward this peak, SPITFIRE therefore simulates more human‐caused ignitions, resulting in higher burned area projections. The importance of human ignitions for future fire regimes in Central Europe has also been recently highlighted by other studies (Berčák et al. 2024; Horn et al. 2024). Beyond Central Europe, population density exerted a notable influence in Northern Europe and parts of the British Isles. In those regions where population density is currently low (e.g., below ~14 individuals per km^2^, Figure S9), declining population density in the future implies fewer human‐caused ignitions (Figures 2 and S12) and explains the decreasing burned area simulated there by SPITFIRE (Figure 4A,B). These results illustrate the important role of population density in SPITFIRE. Population density is also an important driver in other global fire models (Teckentrup et al. 2019), but one global parameterization, as here in SPITFIRE, is unlikely to cover regional specifics, such as here for Europe. The regionally‐calibrated results from BASE might, therefore, be regarded as more realistic to capture human influence on fire ignition and burned area in Europe.

### 4.4 Land‐Use Change

Holding land use constant from 2000 onward (simulations B‐LU & S‐LU, Table 2) had little effect on pan‐European annual burned area in either model (Figure S13). This limited sensitivity likely reflects the small magnitude of land‐use change represented at the continental scale, as indicated by the near‐zero net conversions between forest and grassland (Figures S3 and S4). Such limited change is expected, since major future land‐use expansions are projected to occur primarily in developing regions outside Europe (Popp et al. 2017). However, land‐use changes notably affected burned area in southern Spain and in the Wallachian/Romanian Plain under SSP1‐2.6 in both models (Figures S16 and S17). Both BASE and SPITFIRE respond similarly to land‐use changes, which is consistent with the structure of the LPJmL framework: in this setup, land use primarily affects the extent of burnable area rather than fire behaviour per se. Consequently, the same regional effects appear in both models. Overall, this indicates that land‐use change might be a secondary driver at the continental scale, but can substantially influence regional fire patterns.

Within the SSP1‐2.6 sustainability narrative, projected land abandonment and conversion to natural land in parts of southern and eastern Europe increased the extent of burnable area SSP1‐2.6 (Figure S3B). These changes were associated with the expansion of early‐successional, grass‐dominated vegetation (Figure S10B), which enhanced fine fuel availability and led to higher burned area in both fire models (see Romanian Plain and southern Spain in Figures S16A,C and S17A,C). These findings emphasize a potential trade‐off within SSP1‐2.6: If land is released to meet sustainability objectives such as higher demand for forest products under SSP1‐2.6, wildfires might increase (Preinfalk and Handmer 2024). Regenerating natural areas may contain fire‐prone herbaceous vegetation, as observed in our results for parts of the Balkan region (Figure S10B) that could lead to higher burned area if not anticipated. This underscores the importance of wildfire management in regions experiencing land abandonment or reforestation (Neidermeier et al. 2025).

Under SSP3‐7.0, land‐use changes resulting in increased burnable land were less pronounced than under SSP1‐2.6 (Figure S3C). Simulations with constant land use under this scenario showed only marginal differences compared to simulations with changing land use (Figure S16D vs. Figure 4B), suggesting that worsening fire weather may be a more dominant driver than land‐use change under this high emission scenario. However, in this study, land‐use projections were only feasible until 2050, limiting the representation of longer‐term land‐use dynamics. Beyond mid‐century, land‐use change associated with restoration and biodiversity objectives under SSP1‐2.6 could plausibly intensify, potentially elevating wildfire risk if the fire implications of regenerating landscapes are not adequately anticipated. Under SSP3‐7.0, weak governance and limited land‐use planning may instead promote land abandonment and fuel accumulation beyond 2050. If such land‐use dynamics were to coincide with the pronounced worsening of fire weather projected under SSP3‐7.0, their combined effects could lead to substantially higher burned area than indicated by the current simulations.

Overall, land‐use change has little effect on pan‐European burned area but can substantially influence regional fire activity, particularly where land is abandoned.

### 4.5 Climate‐Induced Vegetation Changes

Climate‐induced vegetation changes alter fuel composition by modifying grass and tree cover, thereby influencing potential burned area. Climate change promotes tree expansion into higher‐elevation areas outcompeting grasses (Figures S10 and S11), while grasses may themselves expand into areas that were previously unvegetated as seen in our simulations for both Scandinavia and the Alps (Figure S10). Despite these vegetation shifts, fire activity remains minimal in Scandinavia due to the very low frequency of ignitions and relatively mild fire weather conditions, resulting in no direct impact of changes in grass cover on burned area (Figure 4, areas with no fire). In the Alps, however, fire dynamics are shaped by contrasting influences: In SPITFIRE, burned area decreases (Figure 4A,B) due to reduced grass cover and increased tree cover that is slowing down fire spread (Figures S10 and S11, Panel B and C respectively). However, in the BASE simulations, Alpine areas often show either increased fire activity or little change, without any consistent spatial pattern (Figure 4C,D).

Additional correlation analyses indicate that the influence of vegetation on burned area is generally weaker and more spatially heterogeneous than the influence of fire weather. Across Europe, correlations between annual burned area and vegetation carbon or fuel load are typically low (between −0.25 and 0.25, see Figures S21 and S22) and show substantial regional variability. In SPITFIRE, vegetation‐related correlations become somewhat stronger under SSP3‐7.0 than under SSP1‐2.6, suggesting that stronger climate change increasingly alters vegetation composition and fuel characteristics in ways that affect fire behaviour. Negative correlations between vegetation variables and burned area are particularly common in Mediterranean regions, indicating that increases in vegetation biomass do not necessarily translate into greater fire activity. Positive correlations occur primarily in areas experiencing natural vegetation recovery following land abandonment, especially under SSP1‐2.6. These patterns highlight the complex and region‐specific role of vegetation in shaping fire activity compared with the more spatially consistent influence of fire weather.

These contrasting results can be attributed to the models' varying sensitivities to fuel composition: SPITFIRE calculates burned area using the Rothermel equation, which explicitly incorporates fuel characteristics such as type, load, and moisture (Rothermel 1972). This mechanistic approach allows SPITFIRE to respond directly to changes in vegetation composition and structure as simulated by the dynamic vegetation in LPJmL. By comparison, BASE captures fuel properties only indirectly, primarily through tree cover, which ranks relatively low among its predictors (fifth place; Forrest et al. 2024). As a result, BASE may be less sensitive to fine‐scale variations in fuel composition. This fundamental difference in how the two models represent fuels likely contributes to the contrasting patterns of fire activity observed in the Alpine regions, with SPITFIRE responding more strongly to changes in vegetation than BASE. Uncertainties in LPJmL's vegetation and fuel representation further affect simulated fire activity. The model does not include managed forests or even‐aged plantations, which are often more fire‐prone, potentially leading to underestimation of burned area (Zald and Dunn 2018; Lindenmayer et al. 2023; Bousfield et al. 2025). Conversely, fuel loads in natural vegetation may be overestimated, as real‐world management such as fuel removal or prescribed burning can reduce available fuels (Neidermeier et al. 2023). Improving the representation of vegetation and fuels could therefore either increase or decrease projected burned area, depending on local conditions and management practices.

In summary, climate‐induced vegetation changes affect fuel composition and regional fire activity, but their impacts are spatially heterogeneous and generally weaker than those associated with fire weather. Nevertheless, vegetation dynamics can substantially modify local fire responses, particularly in regions undergoing pronounced shifts in vegetation composition or land use.

### 4.6 Comparison With Previous European Wildfire Projections

Previous studies have consistently identified climate change as the primary driver of increasing wildfire activity across Europe, particularly under stronger warming scenarios, but they differ in the magnitude and spatial structure of projected changes (Amatulli et al. 2013; Migliavacca et al. 2013; Wu et al. 2015; Khabarov et al. 2016; Turco et al. 2018). Turco et al. (2018) show that burned area increases systematically with warming in Mediterranean Europe, while also arguing that vegetation‐mediated feedbacks and adaptation processes can reduce—but not eliminate—the sensitivity of fire activity to climate change (Turco et al. 2018). In contrast, process‐based DGVM‐fire modelling studies such as Migliavacca et al. (2013) highlight the role of vegetation productivity in constraining fire spread, leading to more moderate or spatially heterogeneous climate sensitivities depending on how vegetation–fire feedbacks are represented (Migliavacca et al. 2013). Similarly, Wu et al. (2015) demonstrate that different DGVM–fire model combinations can produce contrasting regional patterns of future burned area, including both increases and decreases within the same geographic domain, largely driven by differences in how fuels, fire feedbacks, and vegetation dynamics are implemented (Wu et al. 2015). Despite these methodological differences, a common signal emerges across studies: increasing fire weather under climate change consistently drives higher fire activity, while vegetation dynamics and human influences primarily modulate the magnitude and spatial expression of these changes.

Our results are consistent with this broader picture. We further extend it by explicitly incorporating socio‐economic welfare through the BASE model and by contrasting them with a process‐based fire representation in SPITFIRE. The strong influence of the HDI in BASE demonstrates that socio‐economic factors can substantially modify projected fire activity, in some regions even outweighing biophysical drivers under moderate climate change. This suggests that future projection studies would benefit from more explicit integration of socio‐economic processes (such as a proxy for fire management capacity), alongside climate and vegetation dynamics, in order to better capture the full range of mechanisms controlling future fire regimes in Europe.

## 5 Conclusion

In this study, we projected potential future changes in burned area in Europe using two wildfire modelling approaches and two socio‐economic pathways under different climate forcing scenarios. By comparing simulations and analysing how climatic and socio‐economic conditions evolve over time, we identified fire weather and socio‐economic development as the two primary continental‐scale drivers of future fire activity, with vegetation and land‐use effects playing a secondary but regionally important role, particularly under milder fire weather conditions.

Overall, our results are consistent with previous studies identifying climate change as the dominant driver of increasing wildfire risk across Europe, particularly in Mediterranean and other fire‐prone regions. At the same time, our comparison of a process‐based and a socio‐economic statistical model shows that human development could substantially modify the magnitude and spatial expression of future fire activity, depending on the climate forcing level and modelling representation. We further emphasize that the mitigating effect of socio‐economic development on fire activity strongly depends on continued policy decisions and institutional investment in fire management, rather than emerging automatically from development alone. Future wildfire risk will be shaped not only by climate and socio‐economic development, but also by whether governance and fire‐management systems actively adapt and maintain their effectiveness as fire weather intensifies.

These findings highlight that future wildfire regimes in Europe depend on the interaction between climate change and societal adaptation. Under moderate warming, strengthened fire management has the potential to offset part of the climate‐driven increase in burned area, provided that institutional capacity and policy commitment are maintained. Under stronger warming, however, increasingly severe fire weather is likely to exceed the mitigating potential of fire management alone, underscoring that climate mitigation and adaptation are complementary rather than interchangeable strategies for reducing future wildfire risk.

## Author Contributions

**Luke Oberhagemann:** methodology, writing – review and editing. **Christoph Müller:** methodology, writing – review and editing. **Simon P. K. Bowring:** conceptualization, writing – review and editing. **Matthew Forrest:** conceptualization, methodology, writing – review and editing. **Susanne Rolinski:** methodology, writing – review and editing. **Alex N. Neidermeier:** writing – review and editing. **Thomas Hickler:** supervision, funding acquisition, conceptualization, writing – review and editing. **Maik Billing:** conceptualization, writing – original draft, writing – review and editing, visualization, formal analysis, investigation, methodology. **Kirsten Thonicke:** supervision, funding acquisition, conceptualization, writing – review and editing. **Jessica Hetzer:** writing – review and editing. **Werner von Bloh:** writing – review and editing, investigation, methodology.

## Funding

This project has been granted funding from the European Union’s Horizon 2020 research and innovation programme under the Grant Agreement no. 101003890, the Ministry of Research, Science and Culture (MWFK) of Land Brandenburg (22‐Z105‐05/002/001) and Deutsche Forschungsgemeinschaft (GRK 2043/2).

## Conflicts of Interest

The authors declare no conflicts of interest.

## Supporting information


**Figure S1:** Annual burnt area for the SPITFIRE simulations (green), BASE simulations (blue) and the satellite‐based GFED4s product (red). Model simulations show coherent fluctuations compared to observations despite differences in magnitude.
**Figure S2:** Mean fraction of grid cell burnt of the satellite‐based GFED4s product (Panel A), SPITFIRE simulations (Panel B) and BASE (Panel C) – and difference maps in panels D and E. Both modelling approaches also show high burnt area in Southern Europe as the observations, but generally overestimate burnt area in Southern Spain while underestimating wildfires in Northern Portugal.
**Figure S3:** Present‐day proportion of natural land (forests & natural grassland) per grid cell averaged between 2000 and 2010 (Panel A) and average relative changes of natural land until the end of the century (2090–2100) for SSP1‐2.6 (Panel B) and SSP3‐7.0 (Panel C).
**Figure S4:** Present‐day proportion of managed grasslands (pastures) per grid cell averaged between 2000 and 2010 (Panel A) and average relative changes of managed grassland until the end of the century (2090–2100) for SSP1‐2.6 (Panel B) and SSP3‐7.0 (Panel C).
**Figure S5:** Present‐day Canadian Fire Weather Index (FWI) averaged between 2000 and 2030 (Panel A) and average relative changes of FWI until the end of the century (2070–2100) for SSP1‐2.6 (Panel B) and SSP3‐7.0 (Panel C).
**Figure S6:** Annual averaged Canadian Fire Weather Index (FWI) across the whole simulation domain and GCMs for SSP1‐2.6 and SSP3‐7.0.
**Figure S7:** Present‐day Human Development Index (HDI) averaged between 2000 and 2030 (Panel A) and average relative changes of HDI until the end of the century (2070–2100) for SSP1‐2.6 (Panel B) and SSP3‐7.0 (Panel C).
**Figure S8:** Average Human Development Index (HDI) across the simulation domain for SSP1‐2.6 and SSP3‐7.0.
**Figure S9:** Present‐day population density averaged between 2000 and 2030 (Panel A) and average relative changes of population density until the end of the century (2070–2100) for SSP1‐2.6 (Panel B) and SSP3‐7.0 (Panel C).
**Figure S10:** Present‐day natural grass cover per grid cell averaged between 2000 and 2030 (Panel A) and average relative changes of natural grass cover until the end of the century (2070–2100) for SSP1‐2.6 (Panel B) and SSP3‐7.0 (Panel C).
**Figure S11:** Present‐day trees cover per grid cell averaged between 2000 and 2030 (Panel A) and average relative changes of tree cover until the end of the century (2070–2100) for SSP1‐2.6 (Panel B) and SSP3‐7.0 (Panel C).
**Figure S12:** Present‐day rate of human‐caused ignitions averaged between 2000 and 2030 in the SPITFIRE model (Panel A) and average relative changes until the end of the century (2070–2100) for SSP1‐2.6 (Panel B) and SSP3‐7.0 (Panel C).
**Figure S13:** Averaged annual sum of burned area simulated by SPITFIRE (Panel A and B) and BASE (Panel C and D) under SSP1‐2.6 (Panel A and C) and SSP3‐7.0 (Panel B and D). Black lines illustrate simulations where all inputs were allowed to vary; green lines simulations with constant land use; pink lines with constant population density (both hold constant from year 2000). For both models and scenarios holding land use or population density constant had little effects.
**Figure S14:** Relative changes in simulated burned area between years 2000–2030 and 2070–2100 for BASE under SSP1‐2.6 (panel A, C) and SSP3‐7.0 (panel B, D). The lower panels (C & D) show simulations in which the Human Development Index (HDI) is held constant at year 2000 levels.
**Figure S15:** Relative changes in simulated burnt area between years 2000–2030 and 2070–2100 for SPITFIRE under SSP1‐2.6 (panel A, C) and SSP3‐7.0 (panel B, D). The lower panels (C & D) show simulations in which population density is held constant at year 2000.
**Figure S16:** Relative changes in simulated burnt area between years 2000–2030 and 2070–2100 for SPITFIRE under SSP1‐2.6 (panel A, C) and SSP3‐7.0 (panel B, D). The lower panels (C & D) show simulations in which land use is held constant at year 2000.
**Figure S17:** Relative changes in simulated burnt area between years 2000–2030 and 2070–2100 for BASE under SSP1‐2.6 (panel A, C) and SSP3‐7.0 (panel B, D). The lower panels (C & D) show simulations in which land use is held constant at year 2000.
**Figure S18:** Relative changes in simulated burnt area between years 2000–2030 and 2070–2100 for SPITFIRE under SSP1‐2.6 (panel A) and SSP3‐7.0 (panel B) for each GCM.
**Figure S19:** Relative changes in simulated burnt area between years 2000–2030 and 2070–2100 for BASE under SSP1‐2.6 (panel A) and SSP3‐7.0 (panel B) for each GCM.
**Figure S20:** Inter‐model uncertainty in projected burned area change across Europe. Spatial distribution of the standard deviation of ordinal burned area change classes across five global climate model (GCM) simulations. Panels illustrate uncertainty in SPITFIRE simulations (A and B), in BASE (C and D) and for each SSP scenario (A & C, B & D respectively). Higher values indicate greater disagreement among models in the projected burned area change, while lower values indicate stronger inter‐model agreement.
**Figure S21:** Spatial patterns of Pearson correlation coefficients between annual burned area and annual fire weather index (FWI) (panel A & B), vegetation carbon (panel C & D), and fuel load (panel E & F) across Europe for SPITFIRE. Correlations were calculated at each grid cell using annual values over the full simulation period for each GCM separately.
**Figure S22:** Spatial patterns of Pearson correlation coefficients between annual burned area and annual fire weather index (FWI) (panel A & B), vegetation carbon (panel C & D), and fuel load (panel E & F) across Europe for BASE. Correlations were calculated at each grid cell using annual values over the full simulation period for each GCM separately.

## Data Availability

The data that support the findings of this study are openly available in Zenodo at https://doi.org/10.5281/zenodo.18740007.

## Endnotes

^1^

https://forest‐fire.emergency.copernicus.eu/apps/effis.statistics/estimates/ESP.
