# Supplementary material for: Future Projections of Burned Area in Europe Highlight the Importance of Human Action
Source: Glob Chang Biol. 2026 Aug 20;32(8):e71043. doi: 10.1111/gcb.71043 (PMC13491018; doi:10.1111/gcb.71043)
Supplement: Supplementary file 1 [file GCB-32-e71043-s001.zip › Billing_et_all_fullMS_revision_resubmitted_supplement.docx]

# 1. Appendix

## 1.1 Model validation

To evaluate the plausibility of our results, we conducted two reference simulations using historical ERA5-LAND climate data and compared our model outputs with satellite observations from GFED4s. As shown in Figure S 1, the model simulations exhibit coherent temporal fluctuations with the observations. However, regional differences in burnt area estimates were observed (Figure S 2). In the Balkans, the BASE integrated approach overestimated burnt area, whereas the SPITFIRE simulations projected lower values in this region. Both modelling approaches struggled to capture the high burnt area observed in Northern Portugal, though the BASE integrated approach showed slightly better agreement. Outside of Portugal, both models overestimated fire activity in Southern Spain. This discrepancy may stem from fire management responsibilities shifting from national to regional authorities in Spain in the 1990s and 2000s (Moreno et al., 2014), enabling a faster response from firefighting resources - an aspect not directly captured by either model.
In summary, we conclude that both model approaches are capable of adequately simulating wildfires on the European scale with reasonable accuracy in terms of general trends and temporal patterns. While their performance in specific regions - especially in the Iberian Peninsula - must be handled with caution, both models can provide valuable insights into large-scale wildfire trends.

Figure S 1: Annual burnt area for the SPITFIRE simulations (green), BASE simulations (blue) and the satellite-based GFED4s product (red). Model simulations show coherent fluctuations compared to observations despite differences in magnitude.

Figure S 2: Mean fraction of grid cell burnt of the satellite-based GFED4s product (Panel A), SPITFIRE simulations (Panel B) and BASE (Panel C) – and difference maps in panels D and E. Both modelling approaches also show high burnt area in Southern Europe as the observations, but generally overestimate burnt area in Southern Spain while underestimating wildfires in Northern Portugal.

## 1.2 Changes in drivers

### 1.2.1 Changes in land-use

Figure S 3: Present-day proportion of natural land (forests & natural grassland) per grid cell averaged between 2000 and 2010 (Panel A) and average relative changes of natural land until the end of the century (2090-2100) for SSP1-2.6 (Panel B) and SSP3-7.0 (Panel C).

Figure S 4: Present-day proportion of managed grasslands (pastures) per grid cell averaged between 2000 and 2010 (Panel A) and average relative changes of managed grassland until the end of the century (2090-2100) for SSP1-2.6 (Panel B) and SSP3-7.0 (Panel C).

### 1.2.2 Changes in fire weather

Figure S 5: Present-day Canadian Fire Weather Index (FWI) averaged between 2000 and 2030 (Panel A) and average relative changes of FWI until the end of the century (2070-2100) for SSP1-2.6 (Panel B) and SSP3-7.0 (Panel C).

Figure S 6: Annual averaged Canadian Fire Weather Index (FWI) across the whole simulation domain and GCMs for SSP1-2.6 and SSP3-7.0.

### 1.2.3 Changes in HDI

Figure S 7: Present-day Human Development Index (HDI) averaged between 2000 and 2030 (Panel A) and average relative changes of HDI until the end of the century (2070-2100) for SSP1-2.6 (Panel B) and SSP3-7.0 (Panel C).

Figure S 8: Average Human Development Index (HDI) across the simulation domain for SSP1-2.6 and SSP3-7.0.

### 1.2.4 Changes in population density

Figure S 9: Present-day population density averaged between 2000 and 2030 (Panel A) and average relative changes of population density until the end of the century (2070-2100) for SSP1-2.6 (Panel B) and SSP3-7.0 (Panel C).

## 1.3 Changes in vegetation

Figure S 10: Present-day natural grass cover per grid cell averaged between 2000 and 2030 (Panel A) and average relative changes of natural grass cover until the end of the century (2070-2100) for SSP1-2.6 (Panel B) and SSP3-7.0 (Panel C).

Figure S 11: Present-day trees cover per grid cell averaged between 2000 and 2030 (Panel A) and average relative changes of tree cover until the end of the century (2070-2100) for SSP1-2.6 (Panel B) and SSP3-7.0 (Panel C).

## 1.4 Changes in the rate of human-caused ignitions

Figure S 12: Present-day rate of human-caused ignitions averaged between 2000 and 2030 in the SPITFIRE model (Panel A) and average relative changes until the end of the century (2070-2100) for SSP1-2.6 (Panel B) and SSP3-7.0 (Panel C).

## 1.5 Factorial simulations with constant socio-economic drivers

### 1.5.1 Annual burnt area

Figure S 13: Averaged annual sum of burned area simulated by SPITFIRE (Panel A and B) and BASE (Panel C and D) under SSP1-2.6 (Panel A and C) and SSP3-7.0 (Panel B and D). Black lines illustrate simulations where all inputs were allowed to vary; green lines simulations with constant land use; pink lines with constant population density (both hold constant from year 2000). For both models and scenarios holding land use or population density constant had little effects.

### 1.5.2 Spatial relative changes in burned area

Figure S 14: Relative changes in simulated burned area between years 2000 – 2030 and 2070 – 2100 for BASE under SSP1-2.6 (panel A, C) and SSP3-7.0 (panel B, D). The lower panels (C & D) show simulations in which the Human Development Index (HDI) is held constant at year 2000 levels.

Figure S 15: Relative changes in simulated burnt area between years 2000 – 2030 and 2070 – 2100 for SPITFIRE under SSP1-2.6 (panel A, C) and SSP3-7.0 (panel B, D). The lower panels (C & D) show simulations in which population density is held constant at year 2000.

Figure S 16: Relative changes in simulated burnt area between years 2000 – 2030 and 2070 – 2100 for SPITFIRE under SSP1-2.6 (panel A, C) and SSP3-7.0 (panel B, D). The lower panels (C & D) show simulations in which land use is held constant at year 2000.

Figure S 17: Relative changes in simulated burnt area between years 2000 – 2030 and 2070 – 2100 for BASE under SSP1-2.6 (panel A, C) and SSP3-7.0 (panel B, D). The lower panels (C & D) show simulations in which land use is held constant at year 2000.

### 1.5.3 Detailed analysis of GCMs and additional drivers

Figure S 18: Relative changes in simulated burnt area between years 2000 – 2030 and 2070 – 2100 for SPITFIRE under SSP1-2.6 (panel A) and SSP3-7.0 (panel B) for each GCM.

Figure S 19: Relative changes in simulated burnt area between years 2000 – 2030 and 2070 – 2100 for BASE under SSP1-2.6 (panel A) and SSP3-7.0 (panel B) for each GCM.

Figure S 20: Inter-model uncertainty in projected burned area change across Europe. Spatial distribution of the standard deviation of ordinal burned area change classes across five global climate model (GCM) simulations. Panels illustrate uncertainty in SPITFIRE simulations (A and B), in BASE (C and D) and for each SSP scenario (A & C, B & D respectively). Higher values indicate greater disagreement among models in the projected burned area change, while lower values indicate stronger inter-model agreement.

Figure S 21: Spatial patterns of Pearson correlation coefficients between annual burned area and annual fire weather index (FWI) (panel A & B), vegetation carbon (panel C & D), and fuel load (panel E & F) across Europe for SPITFIRE. Correlations were calculated at each grid cell using annual values over the full simulation period for each GCM separately. Results are under SSP1-2.6 (panels A, C, E) and SSP3-7.0 (B, D, F). Positive values indicate that increases in the respective variable are associated with increased burned area, while negative values indicate the opposite relationship.

Figure S 22: Spatial patterns of Pearson correlation coefficients between annual burned area and annual fire weather index (FWI) (panel A & B), vegetation carbon (panel C & D), and fuel load (panel E & F) across Europe for BASE. Correlations were calculated at each grid cell using annual values over the full simulation period for each GCM separately. Results are under SSP1-2.6 (panels A, C, E) and SSP3-7.0 (B, D, F). Positive values indicate that increases in the respective variable are associated with increased burned area, while negative values indicate the opposite relationship.

## 1.6 References

Moreno, M. V., Conedera, M., Chuvieco, E., & Pezzatti, G. B. (2014). Fire regime changes and major driving forces in Spain from 1968 to 2010. *Environmental Science & Policy*, *37*, 11–22. https://doi.org/10.1016/J.ENVSCI.2013.08.005
